# Artificial Warts

**Published:** 1846-04

**Authors:** 


*Artificial Warts.*—A tribe of Negroes inhabiting a part of Africa, on the coast between the Nyambara and Nyango rivers, in about lat. 24 deg. 5′, says Commodore Wilkes, of the Exploring Expedition, possess an art of raising prominent, fleshy tubercles or warts, that is quite mysterious to physiologists. The distinctive personal mark of this tribe, says Mr, Wilkes, is the most extraordinary of any (others, their neighbors, having lines and tattoes;) it consists of a row of artificial pimples or warts, about the size of a pea, beginning in the middle of the upper part of the forehead, and descending to the tip of the nose. Of these they are very proud. Of the manner in which these singular elevations were produced, he continues, we were not able to learn. The slaves whom he examined at Rio Janeiro, having the symmetrical warts, appeared to be averse to speaking of the subject. Take it all in all, the process by which fleshy prominences are developed at will, to remain in a healthy condition through an entire life, baffles conjecture.—*Ibid.*

